# Maximizing Use of Pelagic Capture Fisheries for Global Protein Supply: Potential and Caveats Associated with Fish and Co‐Product Conversion into Value‐Add Ingredients

**DOI:** 10.1002/gch2.202200098

**Published:** 2023-01-20

**Authors:** Maria Hayes

**Affiliations:** ^1^ Food BioSciences Department Teagasc Food Research Centre Dublin 15 Ashtown Ireland

**Keywords:** capture fisheries, diabetes, functional foods, heart health, hydrolysis, pelagic fish

## Abstract

Globally, capture fisheries contribute significantly to protein supply and the food security of a third of the world's population. Although capture fisheries production has not significantly increased in tonnes landed per annum during the last two decades (since 1990), it still produced a greater tonnage of protein than aquaculture in 2018. Policy in the European Union and other locations favors production of fish through aquaculture to preserve existing fish stocks and prevent extinction of species from overfishing. However, aquaculture production of fish in order to feed the growing global population would need to increase from 82 087 kT in 2018 to 129 000 kT by 2050. The Food and Agriculture Organization states that global production of aquatic animals was 178 million tonnes in 2020. Capture fisheries contributed 90 million tonnes (51%) of this. For capture fisheries to be a sustainable practice in alignment with UN sustainability goals, ocean conservation measures must be followed and processing of capture fisheries may need to adapt food‐processing strategies already used extensively in the processing of dairy, meat, and soy. These are required to add value to reduced fish landings and sustain profitability.

## Graphical abstract

Capture fisheries contribute significantly to protein supply and the food security. Less than 1% of total global greenhouse gases result from capture fishery activities. Pelagic fish are nutritious, rich in easily digestible and bioavailable proteins and bioactive peptides. The production of fish protein hydrolysates, their health benefits, and caveats concerning their production are discussed.

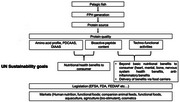

## 1 Introduction

Approximately 7135 kT of crude protein results from harvest of capture fisheries every year^[^ ^1^ ^]^ used for food, fishmeal, and animal feed production. Indeed, the Food and Agriculture Organization (FAO) estimated that capture fisheries contributed 17% of global food protein for human consumption and nearly 20% for two fifths of the global population.^[^ ^2^ ^]^ More recently, the FAO stated that global production of aquatic animals was 178 million tonnes (figure for 2020) and capture fisheries contributed 90 million tonnes (51%). 112 million tonnes was harvested in marine waters, 70% of which came from capture fisheries (FAO, 2022). In addition, according to the FAO, the largest, global producers of fish through capture fisheries and aquaculture combined were China (292 different fish species produced by aquaculture), Indonesia (shrimp, tilapia, and catfish), India (tilapia, shrimp, prawns, and pink perch), Vietnam (pangasius, shrimp, and tilapia), Peru (“anchoveta” (*Engraulis ringens*)) and the EU‐28 (now EU‐27)^[^ ^3^, ^4^ ^]^). In Europe, Russia, Norway, and Iceland dominate production with Russia harvesting Alaskan Pollock; Norway focused on aquaculture production of salmon and capture of pelagic species herring (*Clupea harengus*), blue whiting (*Micromesistius poutassou*), and mackerel (*Scomber scombrus*); and Iceland harvesting blue whiting and cod. The EU‐27 harvests pelagic and demersal fish primarily, and the UK exit from the European Union (EU) in December 2020 (Brexit) and a re‐order of EU/UK trade and cooperation agreements (TACs) due to Brexit along with subsequent EU perception of the UK as “Perfide Albion” has had a significant impact on EU pelagic fish processors. For example, in Ireland, it has resulted in a 15% loss of annual quota mainly affecting the pelagic sector.^[^ ^5^ ^]^

The role of climate change on fisheries sustainability and vice versa, and subsequently, sustainable fish and co‐product supply are critical foundation stones for the development of fish protein hydrolysate (FPH) production. Careful management of fish reserves, sustainable harvest practices, and total utilization of fish landings affect sustainability. Recently, Sandison and colleagues (Sandison et al., 2021) carried out an assessment of the contribution of pelagic fisheries in Scotland to the carbon footprint (CF) and environment.^[^ ^6^ ^]^ In general, pelagic fisheries using the purse seining capture method are fuel efficient, and mid‐trawl fishing was identified as being comparable previously to the purse seining method in terms of fuel efficiency.^[^ ^7^ ^]^ Sandison and colleagues found that pelagic fisheries in Scotland that focus on herring, mackerel, and blue whiting have, overall, a low CF when compared to farmed salmon, shellfish, and demersal fisheries. A standardized attributional life cycle assessments method was used to determine these findings. Moreover, Garnett and colleagues identified that seafood products have a lower CF than animal proteins, and Newton recently identified that protein hydrolysates derived from fish such as blue whiting produce less CO_2_ eq. compared to the production of pea and soy protein concentrates.^[^ ^8^ ^]^

Pelagic fish including blue whiting (*M. poutassou*), mackerel (*S. scombrus*), herring (*C. harengus*), and horse mackerel or scad (*Trachurus trachurus*) are small in size and known to be rich in lipids and poly‐unsaturated fatty acids (PUFAs) including eicosapentaenoic acid (EPA) and docosahexaenoic acid (DHA).^[^ ^9^ ^]^ They are also a source of digestible protein with percentage protein contents ranging from 16% to 20% on a wet weight basis. Blue whiting is normally sold whole frozen. Mackerel and herring may produce fillets—butterfly fillets or flaps and co‐products or side streams from this process step include backbones, heads, viscera, belly flaps, and tails. Co‐products from mackerel and herring processing were previously reported to contain between 37 and 51 g protein/100 g co‐products (head or backbone).^[^ ^10^ ^]^ These co‐products contain a significant source of nutritious protein and represent starting material for the generation of bioactive peptide hydrolysates using different proteolytic enzymes, lactic acid bacteria (LAB), or acids. Other methods for improved utilization of pelagic fish by‐products as human foods include the production of lower‐cost fast foods from trimmings and other by‐products. Traditional ways to utilize by‐products and by‐catch involve isolation of meat and the development of secondary products such as surimi and surimi‐based products, sausages, and fermented products.^[^ ^11^ ^]^ Stevens and colleagues^[^ ^12^ ^]^ recently covered this topic in relation to aquaculture of selected species. Albrektsen and colleagues^[^ ^13^ ^]^ elegantly reviewed the use of marine by‐products for the production of fishmeal and feed for use in aquaculture of salmonids recently. Regardless of the processing method used, the handling of by‐products must be in a food‐grade manner to produce hydrolysates, surimi, or other ingredients suitable for human application. This paper focuses on the use of FPH generation as a strategy to add value to pelagic fishery by‐catch/by‐products. Potential products that result from hydrolysate generation include protein hydrolysates containing bioactive peptides with health benefits, concentrates, oils, calcium, and mineral‐rich bone fractions that may find application in the food, animal, and companion animal fields and functional food markets. These markets are lucrative. For example, the global functional food market was valued at $180.58 billion in 2021 and was projected to grow to $191.68 billion in 2022,^[^ ^14^ ^]^ and the fish protein hydrolysate market was worth $244.2 million in 2021. Although lucrative, these markets are competitive. Within the FPH market, targeted at humans, by‐products from salmon processing are the most established with several key products and players. **Table** **1** highlights existing hydrolysate, oil, and other fish‐derived products sold globally, made from different fish species and co‐products.

**Table 1:** Fish protein hydrolysate and co‐products (oils and minerals) produced from different species including pelagic fish like blue whiting and herring

Company	Fish protein hydrolysate/oil/associated products	Product applications								Species
		Feed	Pet Ingredients		Nutricosmetics		Functional foods/nutraceuticals/supplements	Other	Pharma	Whole blue whiting fish
Bio‐marine Ingredients Ireland (BII Ltd.), Monaghan, Ireland	Fish protein hydrolysates	Yes	Yes		Yes		Yes	Yes	Yes^a)^	Whole blue whiting fish
	ProAtlantic						Fish protein isolate—muscular health; satiety; glycaemic control; weight management			Whole blue whiting fish
	ProShore		Fish protein isolate 55–90% protein pet food applications				ProShore—digestive health in cats			Whole blue whiting fish
	Ishca	Fish protein hydrolysate for hatchery and fish farming—boosts growth								Whole blue whiting fish
	ProGlas							Soil health		Whole blue whiting fish
	Mineral complex (WhiteCal)						Bone health; dental health			Whole blue whiting fish
	Lipids									Whole blue whiting fish
	OmegaBlue						Omega‐3 fish oil powder that guarantees 80 mg EPA g^−1^; 55 g DHA g^−1^			Whole blue whiting fish
Copalis Sea Solutions, France	Fish protein hydrolysates	Yes	Yes		Yes		Yes	Yes	No	Salmon and whitefish
	CPSP	Optimum palatability and contribution to animal well‐being	Optimum palatability and contribution to animal well‐being							Salmon and whitefish
	PHOSCALtm	Fish meal – ideal protein source rich in bioavailable calcium						Fertilizer		Salmon and whitefish
	FISHMEAL	Protein source monogastric animals and used in aquaculture								Salmon and whitefish
	FISHOIL	Use in ruminants, mono‐gastric and aquaculture	Use in pet feeds as a palatant							Salmon and whitefish
	Seanov				Brand of marine ingredients used in functional foods, nutraceuticals, nutricosmetics		Brand of marine ingredients used in functional foods, nutraceuticals, nutricosmetics			Salmon and whitefish
	Collactive				Skin antiaging					Salmon and whitefish
	Collactive HM				Skin antiaging					Salmon and whitefish
	Marine cartilage powder						Bone health; dental health			Salmon and whitefish
	Nutripeptin						Weight control			Salmon and whitefish
	Phoscalim						Bone health; dental health			Salmon and whitefish
	Prolastin				Antiage cosmetic (hydrolyzed elastin)		Joint well‐being			Salmon and whitefish
	Protensin				Inhibits ACE enzyme—heart health					Salmon and whitefish
	PROTEIN M+				Soluble marine cartilage—rich in chondroitin sulfate		Bone health; dental health			Salmon and whitefish
	Protizen						Antistress peptide—well‐being; relaxation—mood food			Salmon and whitefish
	Collagen HM SOL				Pale liquid—moisturizes; stimulates cell regeneration					Salmon and whitefish
	Glycosann sol				Patented chondroitin sulfate with low molecular weight; stimulates the proliferation of fibroblasts and the biosynthesis of collagen and elastin					Salmon and whitefish
	Aromatic extracts									Salmon and whitefish
	Prolys		100% soluble aromatic fish extract —high protein content							Salmon and whitefish
	Profish		Soluble aromatic fish extract							Salmon and whitefish
BASF SE, Brattvåg (Now Marine Ingredients—sold 2014), Norway and Ludwigshafen, Germany. BASF also acquired Pronova in 2013	Highly concentrated omega‐3 fish oils. Products include Omacor (introduced in 1994 as the world's first prescription omega‐3 drug). Pharma products based on omega‐3 from fish oil developed include K85EE omega‐3‐acid ethyl esters (EE); omega‐3‐acid ethyl ester capsules; Maxomega EPA 96/97 EE; CN 600 triglycerides (TG) omega‐3‐acid triglycerides; Maxomega DHA 95 EE AS						Heart health		Omacor is approved for the treatment of hypertriglyceridemia in ≈70 countries worldwide	Salmon
	PronovaPure 360:240 EE						Heart health			Salmon
	PronovaPure360:240 TG						Heart, pregnancy, cognitive, eye health			Salmon
	PronovaPure 150:500 EE						Heart			Salmon
	PronovaPure 46:38 EE						Heart health			Salmon
Croda Healthcare, UK	Incromega DHA–Active Pharma Ingredients (API)						Vision maintenance		Pharma ingredient for vision maintenance	Norwegian sourced fish (likely Salmon)
	Incromega E1070						Eye and brain health		Pharma grade ingredient	Norwegian sourced fish (likely salmon)
	Incromega E550220						Eye and brain health		Pharma grade ingredient	Norwegian sourced fish (likely salmon)
	Incromega TG3322						General well‐being		Pharma grade ingredient	Norwegian sourced fish (likely salmon)
Janatha fish meal and oil products, Kota, India	Fish protein hydrolysates	Yes	Yes		Yes			Yes	No	Varies
	Fish amino acid liquid	Aquaculture applications						Boosts yield of vegetables, fruits and crops		Varies
	Fish amino acid powder							Bio‐stimulant		Varies
	Bone meal							Organic fertilizer		Varies
	Fish meal	Aquaculture applications								Varies
	Fish oil	Animal health–improved immunity against disease, reduced incidences of deformities, higher survival and growth								Varies
	Fish protein hydrolysate liquid (40% hydrolysed protein)	Animal health‐improved immunity against disease, reduced incidences of deformities, higher survival and growth						Feed attractant		Varies
	Fish protein hydrolysate powder (40% hydrolyzed protein)	Animal health—improved immunity against disease, reduced incidences of deformities, higher survival and growth						Feed attractant		Varies
	Sulfited fish oil							Used in leather tanning industry		Varies
	Fat liquors							Used in the tanning process		Varies
	Refined fish oil						Used for cardiovascular health		To make soft gel capsules, hard gelatin capsules,	Varies
Scanbio Marine Group AS, Trondheim, Norway	Fish protein hydrolysate	Yes		Yes		Yes	Yes	Yes	No	Salmon, pelagic, white fish
	ScanPro (salmon, White, Pelagic)	Fish meal replacer—hypoallergenic properties—autolysis process								Salmon, pelagic, white fish
	ScanOil	Animal nutrition								Salmon, pelagic, white fish
	ScanHydro	Animal nutrition								Salmon, pelagic, white fish
	Aquaculture fish silage							Bioenergy		Salmon, pelagic, white fish
Sopropêche, Wimille, France	Fish protein hydrolysate									Salmon, pelagic, white fish
	CPSP 90/CPSP G	Animal nutrition		Pet Feeds/ingredient						Salmon, tuna, trout, herring
	Fishmeal	Animal nutrition		Pet Feeds/ingredient						Salmon, tuna, trout, herring
	Fish oils	Animal nutrition		Pet Feeds/ingredient						Salmon, tuna, trout, herring
Biomega, Bergen, Norway	Fish protein hydrolysates									
	Biomega peptides						Nutraceutical applications; weight control; satiety			Salmon
	Biomega salmon oil						Nutraceutical applications— heart, Body mass index control			Salmon
	Salmigo salmon oil			Nutritional source of protein						Salmon
	Salmigo active			Nutritional source of protein						Salmon
Royal DSM (The Netherlands) Epax AS/Pelagia AS, Bergen, Norway	MEG‐3 fish oils						Provides a source of EPA and DHA			Omega‐3‐rich fish species
	EPA Epax Ultra concentrates and other customized oils								Pharma grade omega‐3 products—guarantee 700 mg g^−1^ EPA/DHA (EPAX Ultra concentrates)	Herring; mackerel
	Enviro fish oil; fish	Aqua and animal diets								
	Group 1 Fishmeal	Good amino acid profile—produced from whole meal pelagic protein (fresh and chilled)—animal/aqua diets								Pelagic fish
	Group 2 Fishmeal	High‐quality protein source—wild caught fish								Wild caught fish
	Group 3 Fishmeal	High‐quality protein produced with low drying temperature—aqua feeds and animal diets								
	Enviro Group 1 Fishmeal	Organic (according to DeBio and Naturland regulations in Norway)—aqua feed and animal diets								Organic fish
	Enviro Group 2 Fishmeal	Animal diets/aqua diets								Organic fish

Bioactive peptides and PUFAs are the building blocks of the fish protein hydrolysate/marine‐derived functional ingredient markets. They are the bioactives responsible for the observed health benefits of commercial fish protein hydrolysates currently available (Table 1) and consist of sequences of amino acids 2–30 in size. Their potential health benefits depend on the inherent amino acid sequences and location of amino acids within the sequence of the peptide. Most bioactive peptides are known to be bioavailable (i.e., cause a health benefit in the body once consumed), as they exist as either pro‐peptides which, following gastrointestinal digestion, may be subsequently cleaved into active, shorter bioactive peptide sequences, sometimes referred to as “cryptides.” Peptides with amino acid sequences less than five amino acids in length can pass through the gut axis to reach their target sites. In addition, the targets for some bioactive peptides may be outside the gut.

Caveats associated with use of hydrolysate products include compliance with different legislation and regulations regarding their marketing and sale in different markets including the EU, USA, Japan, China, and Asia‐Pacific. The European Food Safety Authority (EFSA) in Europe and the Food and Drug Administration (FDA) in America govern human functional foods. Companion animal feed ingredient claims are governed by EU legislation but organizations such as the European Pet Food Industry Federation (FEDIAF) provide nutritional guidelines for complete and complementary pet food for cats and dogs. The FEDIAF also works closely with the American Association of food control officers (AAFCO) in order to keep up to date with the latest recommendations for nutrition and health claims in pets. This paper discusses the potential of pelagic fisheries as a biomass source for protein hydrolysate ingredient generation. It looks at their use in different markets, and it collates details concerning processes and companies currently operating in this sphere. In addition, potential directions that could be pursued to help companies’ transition from production of commodity products to added‐value functional food ingredients for use in different markets are discussed.

## 2 Fish Protein Hydrolysates: Raw Material and Method of Production

### 2.1 Raw Material

Protein hydrolysates can be made from any protein source using enzymatic or acid hydrolysis, high‐pressure processing (HPP), or fermentation with LAB. However, the dry weight (DW) yield of hydrolysate generated from the starting material depends largely on the protein and moisture content of the starting material. Pelagic fish include blue whiting (*M. poutassou*), mackerel (*S. scombrus*), herring (*C. harengus*), and horse mackerel or scad (*T. trachurus*). These fish species have varying content of protein, lipid, moisture, and ash. Blue whiting is harvested from February to March in the North East Atlantic using both trawl and purse sein methods and can contain up to 18% protein and 5% lipids depending on the month of harvest (https://pelagia.com/cm/products/blue-whiting/). It also contains vitamin B12 and selenium. Mackerel contains up to 2670 mg of omega‐3 per 100 g fillet and up to 20% protein containing all essential amino acids. Horse mackerel is harvested in October and November and can contain up to 24% lipid rich in omega‐3, and herring is an excellent source of protein containing about 15.2%. It is also rich in omega‐3. The aim of generating FPHs is to enhance the nutritional and consumer acceptance of fish protein first and second to increase the health benefits and thereby unique selling points (USPs) for fish species. Bioactive peptides are the building blocks of proteins and are the bioactives present in FPHs. They range in size from 2 to 30 amino acids and have a myriad of health benefits as discussed in the following sections. Many are commercialized but not sold for the human functional food market in Europe due to lack of evidence to substantiate health claims. However, they do have markets in the US, Canada, and Asia.

### 2.2 Method of Production of FPH

**Figure 1:**
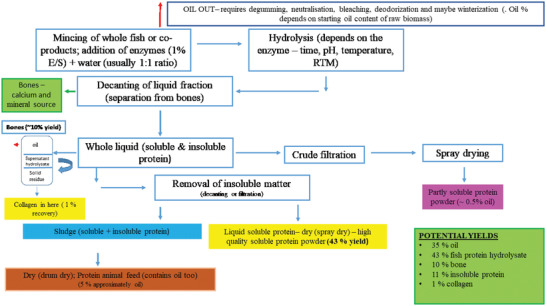
Mass balance indications for fish protein hydrolysate (FPH) generation and recovery of product yields (FPH, collagen, bones, and insoluble material and oil).

FPHs are made by solubilizing the protein present in whole fish or fish co‐products such as skins, heads, tails, fins, or viscera in water using either acids, alkali, or proteolytic enzymes like Alcalase, Papain, Bromelain, Protamex, Endocut, or a combination of two or more enzymes. This process takes place in a bioreactor or hydrolysers, which is a tank equipped with stirrers, a source of heat, and pH control. The temperature, pH, and stirring (revolutions per minute, RPM) conditions are optimized in accordance with the agent used to generate the hydrolysate—in other words, an acid, base, or enzyme source. Proteins present in tissue are solubilized and broken down into smaller proteins and peptides that can range in size from 2 to 30 amino acids in length. The enzyme or acid/alkali is deactivated using either heating or an increase or decrease in pH values, and the degree of hydrolysis (DH) is calculated. The liquid and soluble fractions are separated by filtration, and water is removed using evaporation and drying processes including spray and/or freeze‐drying. The lipid fraction can be separated before or after hydrolysis using centrifugation to remove lipids, and the desired lipid content of any FPH is usually less than 0.5%. Plate and frame filtration processes can be used in combination with micro‐, ultra‐, or nanofiltration. It is important to keep in mind that, over time, filtration columns may clog, and effective cleaning in place (CIP) protocols should be part of any production process. Clogging can result from use of filters with inappropriate pore sizes or pressure, flow rates, pH, and the salt concentration of the solution (Petrova et al., 2018).^[^ ^15^ ^]^ **Figure** **1** shows an overall scheme for production of FPHs. It should be noted that use of co‐products of fish processing such as skins can result in a cleaner end hydrolysate product with uniform peptides due to lack of contamination from other proteins present in the whole fish muscle. Acid and alkali hydrolysate productions have several disadvantages compared to use of proteolytic enzymes. Acid hydrolysis is performed using HCl or sulfuric acid and results in degradation of protein to small peptides and amino acids, and the destruction of tryptophan. Alkali hydrolysis results in the degradation of serine and threonine. Both methods use high temperatures, and disposal of acid/waste by‐products can cause problems. Enzymatic hydrolysis is viewed as a gentle process, and a number of groups have looked at the use of different enzymes applied to either blue whiting, mackerel, or herring raw biomass to date (**Table** **2**).

**Table 2:** Examples of bioactive hydrolysates generated from blue whiting, herring, and mackerel fish species, associated bioactivities and potential health benefits

Starting raw biomass fish type	% DH	Chemical/Enzyme	End product bioactivities or properties	Health/Nutrition impact	References
Isolated protein from whole blue whiting using the pH shift method with 11 m NaOH	2.5%, 5%, 10%, and 15%	Alcalase 2.4 L	Angiotensin‐1‐converting enzyme (ACE) inhibition	Heart health, reduction of hypertension	[16]
Whole blue whiting	<4%	Alcalase 2.4 L and PTN 6.0S	Functional (emulsifying, foaming—hydrolysates with DH less than 4%) antioxidant, ACE‐inhibitory and antigenicity properties	Antioxidant, heart health	[17]
Blue whiting by‐products headed and gutted material	<10%	Acid, alkali, papain	Gelatine extracted—water holding capacity, oil holding capacity assessed	Mouth feel	[18]
Blue whiting	Not given	Flavourzyme and Protamex	Antioxidant activity; protein close to that recommended by WHO	Antioxidant, heart health	[19]
Blue whiting soluble protein hydrolysates	Not given	Not stated	Influence on appetite and weight gain	Reduction of appetite and potentially weight gain	[20]
Frozen headed and gutted hydrolyzed blue whiting proteins	Not given	Alcalase and Protamex	Influence on obesity measured in mice	Reduce obesity	[21]
Atlantic mackerel whole Canadian (collagen fraction from skin)	Not stated	Protamex	Antimicrobial activity against *Staphylococcus aureus*; identification of four bioactive antimicrobial peptides including collagencin from mackerel collagen hydrolysate	Prevention of microbial infection	[22, 23]
Mackerel (*Scomber scombrus*) heads, backbones, and viscera	Not stated	Protease FoodPro PNL	Hydrolysates based on mackerel were slightly more taste intense and had higher scores for umami, salty, and fish taste	Improved taste/umami	[10]
Atlantic mackerel (*S. scombrus*)	Not stated	Protamex	Immunomodulatory peptides	Immune health benefits	[23, 24, 25]
Herring milt	Not stated	Confidential mix of enzymes employed by Ocean NutraSciences, Canada	Changes in gut microbiome that may potentially benefit health; limited reduction in markers for T2D	Potentially, prevention of T2D and improved gut health	[26]
Herring by‐products (head and gonad)	10.1–18.3%	Alcalase	Antioxidant activity; emulsion stability; solubility	Antioxidant health benefits	[27]

### 2.3 Enzyme Choice

Enzyme selection is important as it dictates what peptides will be produced and enzyme selection has an impact on the end FPH in terms of bitterness and sensory characteristics. **Table** **3** highlights commonly used microbial and plant‐derived enzymes used to generate hydrolysates, their optimum pH, temperature, and time, and examples of where they were applied previously to whole fish or fish by‐products. Several companies also have agreements with enzyme suppliers and additionally, by‐products from fisheries including viscera are also being explored as a source of enzymes for use in FPH generation.

**Table 3:** Characteristics of selected microbial and plant origin enzymes used to generate FPHs

Enzyme	Origin	Enzyme	pH	Time	Temperature	Previously generated hydrolysate	Suppliers	References
Alcalase	Microbial—*Bacillus licheniformis*	1–3% of solid biomass	8.5	205 min	55 °C	Originally used in detergent products for removal of protein stains Used previously to generate FPH from eel, beluga, tuna and salmon	Novozyme	[32, 34, 35, 37]
Protamex	Microbial—*Bacillus* sp.	20% of biomass	Neutral	120 min	40 °C	Used previously to generate FPH from beluga (*Huso huso*)	Novozyme	[36]
Neutrase	Microbial—*Bacillus amyloliquefaciens*	1.50%	pH7–9	160 min	60 °C	Tuna by‐product FPH	Novozyme	[34]
Esperase	Microbial—*Bacillus lentus*	0.50%	8.9	80 min	60.8 °C	Catshark (*Scyliorhinus canicular*)	Novozyme	[33]
Papain	Plant origin—*Carica papaya*	3%	7	240 min	40—65 °C	Salmon (Salmo salar); used in dairy production of cheese; used in baking to reduce allergenicity of protein; used in tooth whitening and tissue repair	Merck, Novozyme others	[32]
Bromelain	Plant origin—*Ananas comosus* (stem and juice)	Variable	pH5–10	Variable	70 °C	Used to tenderize meat, generation of FPH, protein degradation in ruminant feed degradation	Merck, several other suppliers	
Ficin	Plant origin—*Ficus carica*	Variable	8	Variable	60 °C	Used to generate FPH from bighead carp (*Hypophthalmichthys nobilis*)	Several global suppliers	[38]
Actinidin KEP40	Plant—Actinidea deliciosa (Kiwi)	Variable	6	Variable	40 °C	Used previously to generate FPH and chicken hydrolyastes and in meat tenderization	Kiwienzyme Limited, New Zealand	www.kiwienzyme.com

### 2.4 Concentration and Drying of FPH

Following hydrolysis, soluble‐protein‐containing material is separated from bone and insoluble material, which may consist of a fat fraction. The fat fraction may be separated before hydrolysis occurs, which allows for recovery of a high‐value oil product rich in omega‐3 PUFAs. A secondary oil fraction may be removed following hydrolysis using either centrifugation or other methods like plate and frame filtration processes (Petrova et al., 2018). Before drying, it is economical to concentrate the FPH by removing water. Evaporators are used for this purpose. Up to 50% solids can be obtained following evaporation processes. Different evaporators exist including falling or rising film evaporators where the hydrolysate mixture is heated and water is removed at either the top or bottom of the evaporator through a valve (Petrova et al., 2018). FPHs are dried using spray driers following the concentration phase. Prior to concentration, hydrolysates can be further concentrated using microfiltration and nanofiltration processes or molecular weight cut off (MWCO) filtration using ceramic membranes to recover proteins/peptides and to enhance the protein concentration of the hydrolysate and remove salt. Bones can also be dewatered and are a valuable source of collagen/gelatine peptides. The moisture content of bones varies and these can be dried using specialist equipment such as Coctio Ltd. bone dryer.

### 2.5 Caveats Regarding Enzyme Use in Pelagic FPH Generation

There are several limitations associated with use of enzymes to generate FPHs. The first is the cost of enzymes in terms of purchase price but also associated costs to consider regarding deactivation costs related to the amount of energy required to stop the enzyme and the duration of time hydrolysis with a given enzyme required.

Use of proteolytic enzymes in hydrolysis of fish biomass enables retention of nutritional value of the source protein and allows for precise hydrolysate production (Zamora‐Sillero et al., 2018).^[^ ^28^ ^]^ At the end of an enzymatic hydrolysis process, there are no organic solvents or chemicals to dispose of. Plant and microbial enzymes often used in hydrolysis production are shown in Table 3.

Other issues include process control, which depends on the enzyme used, and additionally resultant bitterness in the hydrolysate product.^[^ ^29^ ^]^ Previously, Slizyte and colleagues assessed the bitterness of peptides produced using enzymatic treatment and found that the use of enzymes Bromelain and Papain resulted in a less bitter FPH from herring.^[^ ^30^ ^]^ The bitterness of FPH is often due to hydrophobicity, degree of hydrolysis, molecular weight, proline residues, type of enzymes, and amino acid sequences. Peptides with bulky hydrophobic groups toward at the C‐terminal of the peptide may be responsible for bitterness.^[^ ^31^ ^]^ Bitterness may be removed using extraction of bitter peptides with alcohol, use of activated carbon treatment, the Maillard reaction, use of cyclodextrin, chromatographic separation, and enzymatic hydrolysis with exopeptidase, and plastein reactions can remove bitterness at an extra cost. These methods reduce bitterness and may improve FPH taste, but some bioactivities may be lost depending on what peptides are removed using individual processes.^[^ ^32^ ^]^

Results in terms of mass balance, content and molecular weight of peptides, and bitterness are often omitted from papers. However, bioactivities in terms of health benefits are often reported, albeit, studies relate usually to potential health benefits identified using in vitro bioassays or small‐scale animal trials in mice models.

### 2.6 Methods to Improve Production of FPHs: Use of In Silico Strategies

**Figure 2:**
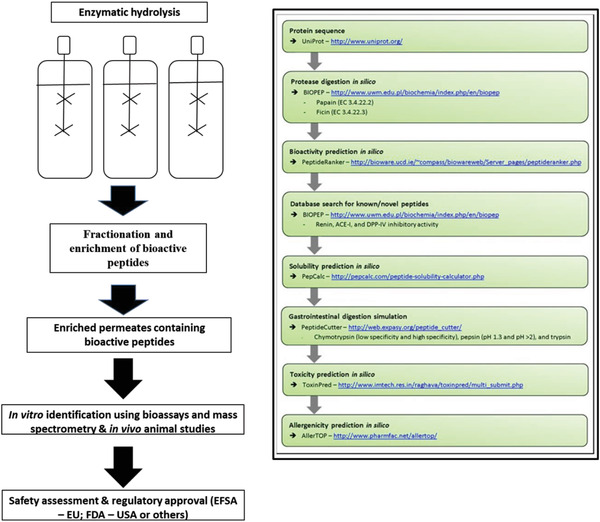
In silico method for generation and identification of bioactive peptide containing pelagic protein hydrolysates.

In silico methodologies can be used to select enzymes for use in the FPH process, aid in vitro bioactivity screening of resultant bioactive peptides, and select hydrolysates for screening in vivo based on peptides identified using mass spectrometry in the discovery stage of functional food ingredient development. Recently, several studies have highlighted the benefits of using in silico methodologies to generate FPHs from shellfish and fish.^[^ ^39^, ^40^ ^]^ **Figure** **2** highlights useful in silico database and methods that can be applied prior to initiation of FPH generation at lab scale, for selection of biomass or enzymes. It also shows how post‐bioactive peptide amino acid sequence identification using mass spectrometry, in silico methods, can be used to identify/assign potential health benefits to the fish hydrolysate. In addition to these methods, multivariate correlation of infrared fingerprints and molecular weight distributions of protein hydrolysates could be used in real time to determine potential bioactivities of hydrolysates as detailed by Nofima for milk and chicken hydrolysates previously.^[^ ^41^ ^]^

### 2.7 Bioactivities and Nutritional Quality of FPHs: Model Selection Matters

The topic of food‐derived bioactive peptides for health is of great interest to industry due to evolving drivers in food product innovation, including health and wellness for the elderly, infant nutrition, and optimum nutrition for sports athletes as well as associated benefits for companion animals/pets. The bioactivities of fish protein hydrolysates generated from pelagic fish species are largely characterized to date using in vitro bioassays. To date, antioxidant, angiotensin 1‐converting enzyme (ACE‐1; EC 3.4.15.1) inhibitory activity, dipeptidyl peptidase IV (DPP‐IV; EC 3.4.14.5) inhibition, and Glucagon‐like peptide 1 inhibition indicate that blue whiting hydrolysates may have a myriad of health benefits (Table 2). Health benefits may include a positive impact on heart health and hypertension, prevention of type‐2 diabetes (T2D), and satiety. However, no studies to date were carried out in human or larger mammal studies beyond mouse models. This is limiting in relation to companies making novel health claims, especially in Europe, related to blue whiting, herring, and mackerel hydrolysates for use as functional foods.

In terms of their use in companion animals, fish protein hydrolysates could provide an excellent source of amino acids and omega fatty acids. Previously, Folador and colleagues (Folador et al., 2006) identified that Pollock milt, red salmon hydrolysate, and smallmouth bass provided the best source of PUFAs.^[^ ^42^ ^]^ Pollock liver and viscera had high total fatty acid and were highly palatable when assessed in a dog feeding trial suggesting that they could make effective palatants in pet foods. It is important to have the chemical composition, protein quality, and palatability tests concluded for any FPH as fish substrates differ significantly and are affected by the fish part used. However, FPHs have potential for use in dry extruded pet products as well as canned foods.

The nutritional quality of FPHs depends on several functional attributes of the hydrolysate including solubility, digestibility, and bioavailability. These are linked to the amino acid profile of the hydrolysate, which depends largely on the source fish protein material used to make the hydrolysate and the DH which depends on enzyme efficiency. In terms of use for human foods, the protein digestibility corrected amino acid score (PDCAAS) value dictates protein quality. This method was replaced by the digestible indispensable amino acid score (DIAAS) method recently by the FAO. The nutritional quality of FPHs depends on the amino acid content and the bioavailability of these amino acids once consumed by a human. A scale of 0–1 indicates protein quality, and the closer the protein is to 1 the better the nutritional value of the protein source. The PDCAAS value of an FPH can be determined in vitro using the Megazyme assay kit as reported recently.^[^ ^43^ ^]^ Several human‐simulated digestion models exist that claim to be able to determine bioavailability and bioaccessibility of proteins including hydrolysates, but how closely these methods or the in vitro Megazyme assay kit methods mimic the real situation in the human gastrointestinal tract is not known. However, a recent study by Mulet‐Cabero and colleagues found that using the International network of excellence on the fate of food in the gastrointestinal tract digestion method, similar scores were obtained for proteins passed through this simulated model in terms of bioavailability compared to proteins trialed using the PDCAAS method in costly rat and mouse models.^[^ ^44^ ^]^

In terms of FPH use in animal, fish, or companion animal models, use of animal models to trial the nutritional benefits of the hydrolysate is preferred. However, for ethical reasons, it is better to use in vitro methods initially to assess FPH quality and in vitro methods to do this include the International Fishmeal and Oil Manufactures Association pepsin digestibility assay (used to determine the quality of fishmeal) and methods such as in vitro static simulated gastrointestinal digestion model adapted to the dogs system. Recently, the promising effects of a tilapia by‐product hydrolysate on the regulation of food intake and glucose metabolism were determined using a simulated dog gastrointestinal digestion model, and new bioactive peptides with antidiabetes/antiobesity benefits were identified.^[^ ^45^ ^]^

## 3 Legislation and Regulations

### 3.1 Human

If sold in the EU27 FPHs for use as nutritional or health beneficial ingredients must comply with relevant regulations including Novel Food Regulation (EU) 2015/2283 and Nutrition and Health Claim regulation (EC) No. 1924/2006 as well as relevant food additive regulations ((EC) 1333/2008) and food contaminant regulation ((EC 1881/2006).

The European Union regulation (EU 2017/2470) maintains the Novel Food Catalogue, which includes all authorized novel foods. The list is nonexhaustive and contains information collected from the EU27. Items consumed commonly prior to 15 May 1997 are not subjected to the novel food regulation. For example, collagen, which can be derived from pelagic fish skins and bones, is listed in the catalog with the description “The request concerns the use of hydrolyzed collagen of animal origin in beverages. Such use is not novel” (https://webgate.ec.europa.eu/fip/novel_food_catalogue/#). Omega‐3 fatty acid lysine salt is also listed in the catalog with the description “The request concerns whether Omega‐3 fatty acid‐lysine salt for use as an ingredient in food supplements falls within the scope of the novel food regulation. The conclusion is that the product is not a novel food. This is on the basis that the food is not a new molecular structure within the meaning of Article 3(2) (2) (a) (i) of Regulation (EU) 2015/2283.” Ethyl esters (EE) concentrated from fish oils are also listed with the description “The long‐chain *n*‐3 polyunsaturated fatty acids (PUFA) are characteristic of marine fat and commonly occur in triacylglycerol's and phospholipids of fish. Effects of marine fat are known and almost exclusively attributed to the most ubiquitous of the *n*‐3 fatty acids in fish which are EPA (C20:5*n*‐3) and DHA (C22:6*n*‐3) both originating from the polyunsaturated α‐linolenic fatty acid (ALA 18:3*n*‐3).” The novel status is listed as FS for ethyl esters meaning that ethyl esters were used prior to 15 May 1997 as or in a food supplement and any other food use of this product have to be authorized pursuant to the Novel Food Regulation. Interestingly, another ingredient listed is a peptide extract from hydrolyzed parts of pacific cod (*Gadus macrocephalus*) and this is considered a novel food. This means that before this product is placed on the market in the EU, a safety assessment under the Novel Food Regulation is required.

In addition to the novel food regulation, the health benefits of FPHs for potential sale in the EU27 is governed by the nutrition and health claims for foods regulation and includes the use of food supplements. The EFSA decides, using a weight of evidence approach if there is enough scientific evidence available to support and grant a nutrition and health claim to a product. Nutrition claims are permitted if they are listed in the Annex.^[^ ^46^ ^]^ Claims such as “contains a source of vitamins or minerals” can be used if the FPH contains 15% of the recommended daily allowance (RDA) of 100 g/100 mL or a portion. RDAs are defined in Annex XIII of the Nutrition Information Regulation (EU) 1169/2011.^[46^ ^]^

Often, FPH occupies the legislative area between foods and cosmetics. In the EU, the Cosmetics Directive defines a cosmetic as “any substance or preparation intended to be placed in contact with the various external parts of the human body or with the teeth and the mucous membranes of the oral cavity with a view exclusively or mainly to cleaning them, perfuming them, changing their appearance and/or correcting body odours and/or protecting them or keeping them in good condition”. However, nutricosmetics are food ingredients or supplements that have a beneficial effect on the external parts of our body by changing their appearance. They are consumed usually as capsules, but are claimed to affect positively the skin. Examples are the products Collactive and Hydro MN—produced through hydrolysis of fish‐derived collagen and elastin protein and claim to have “antiwrinkle action.”^[^ ^47^ ^]^

At present, no pelagic‐derived FPHs have obtained health claims in the EU27, and usually FPH ingredients developed by companies such as those listed in Table 1 are sold outside of the EU27. There is a particularly strong market for FPH in Canada and Asia, for example. In the USA, Canada, Japan, and Asia, legislation and regulation also apply to the sale of any FPH as a nutritional, health, or novel food. The Japanese Ministry of Health, Labour and Welfare (MHLW) introduced the Foods for Specified Health Uses (FoSHU) system in Japan as the approval system for the regulation of all health claims on packages of food products launched in Japan.^[^ ^47^ ^]^ In addition, the FDA and the Nutritional Labelling and Education Act (NLEA), which regulates health claims and food labeling, govern the US procedures.

### 3.2 Companion Animal

Within the EU, use of functional foods for companion animals could see growth in the future due to the limitations regarding prescription drugs for animals under the EU 2019/6 and 2019/4 Veterinary Medicinal Products Regulations that came into effect on 28 January 2022. The FEDIAF represents the European pet food industry. The FEDIAF has a Pet Food Labelling Code that is approved by the EU Standing Committee for Animal Nutrition. This code can guide utilization of FPH as pet ingredients. Several claims regarding ingredients for pets now include an environmental dimension such as “Zero Paw prints by 2050.” Claims such as this must provide evidence of clean label ingredients and sustainability certification from marine bodies such as Marine Trust of Aquaculture Stewardship Council (ASC).

In the USA, it is estimated that 10–33% of dogs and cats are given supplements, a very lucrative market. The FDA, specifically the Centre for Veterinary Medicine (CVM), is responsible for the regulation of animal food products and has procedures to approve food additives for use, which apply to any product unless it is generally recognized as safe for that intended use (i.e., forages, grains, and most mineral and vitamins).^[^ ^48^ ^]^ If an FPH claims: “to cure, treat, prevent, or mitigate disease, the product should be considered a “new animal drug”. Pet food, including pet treats but not pet supplements, falls under the AAFCO and these are regulated at federal and state levels.^[^ ^48^ ^]^ FPHs marketed as nutritional supplements must adhere to set guidelines. They must, for example, state a known need for each nutrient ingredient represented to be in the product. The label of the product can only be used in supplementation and not as a substitute for good daily rations. Thirdly, the product must provide meaningful but not excessive amount of each of the nutrients that it is supposed to contain. Fourthly, the labelling should bear no disease prevention of therapeutic, including growth promotional, representations. The label should not be otherwise false or misleading in any particular, and the product must not be either over‐potent or under‐potent or pose a hazard to the health of the target animal.”^[^ ^48^ ^]^ If producing and marketing a product to improve the health of a companion animal, however, companies can state the word “health” on the label of the product.

## 4 Conclusion

The application of pelagic FPH in functional foods for nutritional and health benefits as well as in feed and treat products for companion animals offers many opportunities and challenges and may help to supply an excellent source of protein to the growing global population. Sustainable supply of pelagic fish is critical to the development of these food and feed ingredients and hydrolysis offers a method to ensure total utilization of pelagic fish catch and generation of ingredients that consumers are likely to accept. The use of mesopelagic fish species for FPH production and PUFA oil production is also a possibility and is being researched currently as part of the ecologically and economically sustainable mesopelagic fisheries (MEESO) project (https://www.meeso.org/). However, the economic cost in terms of capital expenditures for plant establishment to generate pelagic FPH can be considered a limiting factor for some processors currently. The science regarding identification of health benefits of FPHs is growing but there is a need to educate processors regarding the full potential of their pelagic catch. There is also a need to educate consumers regarding the health and nutritional benefits of pelagic fish and to continue to create high‐value functional food products for health. Importantly, fish stocks must be maintained and pelagic biomass used in generation of FPHs much have certification by bodies like ASC or Marin Trust. As described previously by Dragøy‐Whitaker and colleagues^[^ ^49^ ^]^ the use of accessible demonstration plants is a valuable resource for companies/processors to demonstrate what is possible before committing to investment in FPH equipment and plants. A useful resource for companies interested in pursuing commercial production of pelagic FPH is Pilots4U 2020 available at https://biopilots4u.eu/. This website lists available pilot plants to trial FPH production prior to proceeding to commercial scale. Pelagic FPHs have potential to supply high quality, nutritious protein to the global population and enhance the health of communities due to their marine bioactive peptide contents and nutritional benefits. They may form part of a health maintenance/preventative healthcare strategy that could reduce medicine costs for Governments and society.

## Conflict of Interest

The author declares no conflict of interest.

## Data Availability

Data used to write this paper is available from the corresponding author.
